# The shaded side of the UHC cube: a systematic review of human resources for health management and administration in social health protection schemes

**DOI:** 10.1186/s13561-018-0188-4

**Published:** 2018-02-20

**Authors:** Konrad Obermann, Tata Chanturidze, Bernd Glazinski, Karin Dobberschuetz, Heiko Steinhauer, Jean-Olivier Schmidt

**Affiliations:** 1 Mannheim Institute of Public Health (MIPH), Heidelberg University, Ludolf-Krehl-Str. 7-11, Mannheim, 68167 Germany; 2 Oxford Policy Management, Oxford, UK; 3 Rheinische Fachhochschule, Cologne, Germany; 4 AOK International Consulting by KomPart, Berlin, Germany; 5 GIZ Deutsche Gesellschaft für Internationale Zusammenarbeit, Eschborn, Germany

**Keywords:** Human resources, Health financing, Health administration, Health care purchasing, Management, UHC, I11, I13, J24, J45

## Abstract

Managers and administrators in charge of social protection and health financing, service purchasing and provision play a crucial role in harnessing the potential advantage of prudent organization, management and purchasing of health services, thereby supporting the attainment of Universal Health Coverage. However, very little is known about the needed quantity and quality of such staff, in particular when it comes to those institutions managing mandatory health insurance schemes and purchasing services. As many health care systems in low- and middle-income countries move towards independent institutions (both purchasers and providers) there is a clear need to have good data on staff and administrative cost in different social health protection schemes as a basis for investing in the development of a cadre of health managers and administrators for such schemes. We report on a systematic literature review of human resources in health management and administration in social protection schemes and suggest some aspects in moving research, practical applications and the policy debate forward.

## Introduction

The health workforce has received major policy attention over the past decade, not least by the Millennium Development Goals (MDGs) and now the Sustainable Development Goals (SDGs) and universal health coverage (UHC). UHC is today a widely acclaimed conceptual idea to improve access to health services of populations, providing access to quality care while ensuring that there is no major financial risk for patients. After publication of the 2010 World Health Report on health system financing, more than 60 countries have approached WHO for technical support in moving towards universal coverage [5].

Achieving UHC has led to intense technical debates on funding, pooling, purchasing, and the provision of medical staff to deliver such care. The importance of strategic purchasing for improving health sector outcomes and efficiency has been recently highlighted in a number of studies [21]. A health workforce sufficient in numbers, adequately distributed, and well trained and performing is critical for achieving UHC. The Global Health Workforce Alliance has conducted a whole range of country specific analyses on gaps and shortages, [11] but the debate about human resources for health primarily focuses on the availability of clinical staff [10, 23]. These professionals indeed play a key role in delivering quality health care, but they are embedded in, and thus dependent on a web of administrative and management practices in the wider social protection scheme. While there are detailed WHO recommendations for human resources in health (HRH), e.g. the number and profile of clinical staff in different institutions, there is a lack of standards for health management and financing and social health protection expertise.

This article reviews the current knowledge about staff in health care purchasing and management of social health protection schemes in all countries irrespective of their income level. We then suggest some elements of developing this area of research and policy based on a narrative review of relevant studies combined with the management, consulting and field experience of all authors and outline areas of further research.

## Material and methods

We performed a systematic literature research based on the PRISMA approach [15], included PubMed Central, EconLit and Science Direct and used the following search string (full text search):

*(“social health insurance” OR “social security fund”) AND (administration OR administrative OR management OR “health manager” OR public) AND (staff OR workforce OR personnel OR employees OR “human resource” OR “human resource management”)*

To narrow the search and to receive more specific results, the following search string was used for Google Scholar (full text search):

*(“social health insurance” OR “social security funds”) AND (“(administration OR administra- tive) (staff OR workforce OR personnel)” OR “management (staff OR workforce OR person- nel)” OR “health (managers OR administrators)”)*

To cover the last 10 years, the search was restricted to publications since 2007. To focus on social security schemes, this systematic literature research was further restricted to countries whose social security funds accounted for at least 5% of their total health expenditure (based on data from WHO Global Health Expenditure Database) (Table 1).

**Table 1:** The 85 countries for which data an Social Security Funds and THE was available and whose social security funds accounted for at least 5% on their total health expenditure

Income group	Population in millions			
	< 3	3–100		> 100
Low income		• Mozambique • Nepal • Rwanda	• Togo • Zimbabwe	
Lower-middle income	• Cabao Verde • Djibouti • Micronesia • Mongolia	• Bolivia • Egypt • El Salvador • Ghana • Guatemala • Honduras • Kenya • Kyrgyz Republic	• Mauritania • Moldova • Morocco • Nicaragua • Philippines • Tunisia • Vietnam	• Indonesia
Upper-middle income	• Albania • Belize • Gabon • FYR Macedonia • Maldives • Marshall Island • Montenegro • Suriname	• Algeria • Argentina • Bosnia and Herzegovina • Bulgaria • Colombia • Costa Rica • Dominican Republic • Ecuador • Georgia • Iran	• Jordan • Lebanon • Panama • Paraguay • Peru • Romania • Serbia • Thailand • Turkey • Venezuela	• China • Mexico • Russia Federation
High income	• Andorra • Antigua and Barbuda • Estonia • Iceland • Lithuania • Luxembourg • Monaco • San Marino • Slovenia	• Austria • Belgium • Croatia • Czech Republic • Finland • France • Germany • Greece • Hungary	• Israel • Netherlands • New Zealand • Norway • Poland • Slovak Republic • South Korea • Spain • Uruguay	• Japan • United States

Source: Authors, classification of income based on [25]

The literature research was performed on July 12, 2017. During the process of title and abstract screen only references which were likely to contain data on human resources for health in administration were included; studies on health system evaluation and reforms were included as well. All references without relation to health or social security schemes were excluded. Studies dealing with specific diseases or treatments, access to healthcare or population coverage, reimbursement and contributions were also excluded in the first step. Lastly, studies assessing solely and explicitly hospital management issues (i.e. operational management and leadership skills on a management level) and specialities of clinical workforce (i.e. only physicians) were excluded. The full texts of remaining references were read. Only references including either quantitative or qualitative (or both) information on HRH in administration and management were included during this step. Qualitative data was defined as administrative staff descriptions, tasks and activities that are performed. Studies that did not contain data on HRH or solely data on professions other than administration and management (i.e. physicians, nurses or midwives) were excluded.

## Review results and discussion

**Fig. 1:**
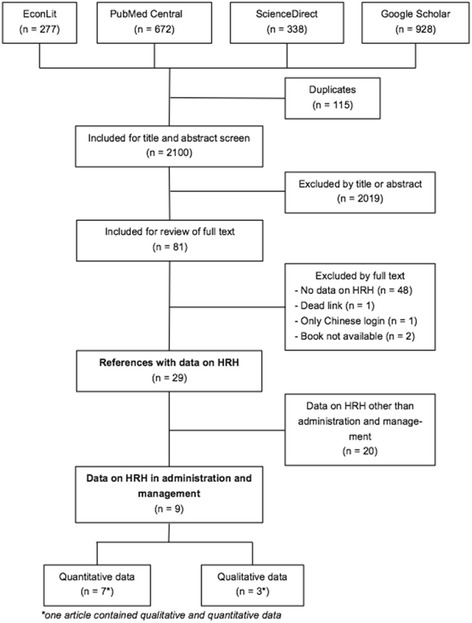
Flow diagram of study selection

Overall, 2215 articles and books were found with the thesis’ search strategy (see Fig. 1). After eliminating duplicates with EndNote X7 and manually, 2100 articles were screened by title and abstract. Of those, 81 articles met the inclusion criteria and their full texts were obtained (not possible for 4 articles, respectively books). Only 29 of these articles contained data on HRH and only 9 on HRH in administration and management. Six articles contained quantitative, two articles qualitative and one article both, qualitative and quantitative, data on HRH in administration and management. Figure 1 shows the flow diagram of study selection.

None of the nine articles tackled the topic of existent or necessary amount of managerial and administrative staff in healthcare directly or comprehensively. The studies focused mainly on reviews and reforms of health systems and institutions (Ministry of Health) [1, 7, 9, 14] or HRH in general [12]. The information on administrative staff was more or less supplementary. The OECD published an article on ineffective use of resources in healthcare systems and presented reasons for high administrative costs and different administrative tasks without providing quantitative data on HRH in administration and management [19]. One study was a proposal for the implementation of a health insurance scheme in Nepal [18]. It contained the proposed organisational structure, the description of administrative roles and calculated staffing numbers (per insured persons) and administrative costs. Of the two studies that addressed management and administration in healthcare issues directly, one analysed the impact of management capacity on the rural New Cooperative Medical Scheme (NCMS) in China on a county level [26]. Even though the focus did no lay on the amount of HRH personnel, they provided figures on the staff in 6 district offices, representing a population of less than 2 million insured persons.

We could only find a single review [16] concerning health management and administration in social protection schemes. It analysed administration costs but also illustrated administrative tasks that need to be performed.

In what follows we provide some theoretical thoughts, based on the review of the (limited) international experience and anecdotal evidence concerning management and purchasing from a social health fund perspective.

### The missing aspect: Health management and administration

In 2010, the WHO presented ‘Monitoring the Building Blocks of Health Systems: A Handbook of Indicators and Their Measurement Strategies’ [24], which mentions selected aspect of management staff. It presents a classification of health workers based on criteria for vocational education and training, regulation of health professions, and activities and tasks of jobs, and draws on the latest revision to the International Standard Classification of Occupations (ISCO). Health management is in a category with janitors and drivers.

A recent book by the World Bank [22] aiming to help decision makers better understand and address their workforce challenges mentions “Leadership, governance, and management weaknesses” (p. 2) and emphasizes the importance of management capacity, but does not cover the management of either health financing and purchasing entities or health facilities.

This lack of coverage might be explained by fundamental differences between clinical HRH and managerial and administrative HRH as part of social protection expertise, as the needs for the former are universally grounded in the bio-medical nature of human beings in terms of prevention, treatment, and rehabilitation, while the needs for the latter are shaped by very specific characteristics of a country’s health and social protection systems based on different institutional developments and societal norms and values. Nevertheless, independent of the country-specific situations, one can define certain minimally required sets of skills, expertise, personnel and institutions needed when moving towards UHC. Comparing tax systems may serve as an analogy here.

### Management and purchasing in health

While many management functions in health are similar to those in other industries, there are specifics in the health care sector which do not allow for a simple application of experience and tools that proved effective elsewhere. Health care is usually an extremely regulated field: this applies to licensing physicians, accrediting hospitals, controlling quality, governing financing and curtailing costs, and claims management. The specific demands implied by such regulation add complexity to the management and administration practice in the health sector. With the rise of mandatory health insurance schemes and dedicated paying institutions within integrated tax-financed health systems, health service purchasing experience is required.

This explicit purchasing function (and not merely a reimbursement of costs) needs a number of key competencies. Based on a fundamental understanding of the national health care policy, laws and regulations as the framework conditions, this includes a good knowledge about the insured population, responsiveness to health needs and preferences, administration of the insured (registration, collecting contributions, information & advice and directing patients, control of fraud and abuse), a good understanding of health technology assessment, assessment of provider competencies (service quality and accreditation, claims management), negotiation with providers (on volume, quality and cost of services) subsequently contracting and monitoring and controlling results. Table 2 below gives an overview of the wide range of competencies needed.

**Table 2:** Core departments und major functions in a public purchasing organization

Organizational unit	Responsibilities
Chief executive	Oversight and overall responsibility
Ombudsman	Independent inquiry of complaints filed
Public relations	Communicating with the public, annual reports Working with parliament and the Ministry of Health Coordinate and exchange with international institutions Responding to objections and comments from the public
Internal auditing	Auditing of operations of all departments and branches Reviewing fraud and corruption risks and whistle-blowing Proposing enhancements for internal operations
Legal affairs	Reviewing supply contracts Reviewing and preparation of all contracts with providers Settlement of legal issues with insurees and providers
Actuary and statistics	Assessing financial impact of changes to the benefit package and health technology assessment Proposing and assessing the impact of cost-sharing schemes Estimating cost implications of epidemiological and demographic trends to the benefit package Estimating cost implications on any policy decision that needs to be reflected in the composition of benefit package Calculation of contribution rates Actuarial/statistical reports
Human resources and training	Recruiting and retention of staff Maintaining personnel records Plan and organize training / educational plans for internal staff
Marketing	Preparation, running, evaluation of marketing campaigns Preparation of information booklets and website Answering queries of insurees, media and civil society
Registration	Collecting forms on enrolment or renewals from field staff Recording data on households/members and sold policies Issuing insurance cards
Service purchasing	Definition of the benefit package, including costing / pricing and health technology assessment Development of remuneration mechanisms for providers Creation and maintenance of classifications Preparation of model contracts for types of providers Negotiating with health care providers on contractual terms Accreditation / maintaining the register of providers Checking the correctness of claims, medical review Communicating with providers on findings and problems
Finance and accounting	Accounting for all financial operations, valuating claims Producing basic accounting reports for the annual report Depositing/investing available funds
IT	Specification of requirements for IT support Design and maintenance of all forms for business processes Communication with IT vendors Running help desk for internal staff
Organizational development	Define the different staff positions regarding the tasks and the corresponding requirements in skills and expertise

Source: Authors, based on [20]

Similarly, enhanced management and administration expertise is needed from the delivery side. Hospital and practice managers need to be well aware of the complex regulatory environment when negotiating with purchasers, managing personnel with a high level of professional autonomy, introducing quality assurance measures while organizing services to effectively and efficiently respond to the population’s needs. Health care service management in LMIC has been well studied [6].

### International comparisons

Mathauer and Nicolle [16] reviewed global health insurance administrative costs. They found huge variations with (i) costs for administering private health insurance about three times higher than those for administering social health insurance, (ii) administrative costs in low- and middle-income countries much higher than in high-income countries and (iii) with considerable variations across and within countries over time. The authors rightly point out that “*simple comparisons of shares of administrative costs are inadequate. There is thus need to look beyond aggregate numbers*.” The findings imply a wide variation in the role and work load of insurance schemes, a lack of standardized processes within the insurances’ administration and possibly insufficient governance and management expertise.

Borghi et al. [2] assessed annual facility and district-level costs of running the Community Health Fund (CHF) in Tanzania. They found that the cost of administering the CHF was very high with a total cost to revenue ratio ranging from 50% to 364% with advertising and revenue collection being the most resource-intensive activities. Interestingly, the authors found that facilities with lower case loads were able to achieve a lower cost to revenue ratio than facilities with higher case loads, indicating dyseconomies of scale.

Yan and colleagues [26] report on a qualitative study about the extent and impact of county level managerial capacity to manage the New Cooperative Medical Scheme in China. They found serious shortcomings concerning staffing, organization and definition of responsibilities in areas such as premium collection and remuneration. In addition, individual counties were restricted in their ability to use resources for management, lacked support from other organizations and suffered from a conflict of responsibilities. The authors point out the need for effective management capacity in handling the scheme and suggest options for content and process of management capacity development.

We did not look at the discussion on health care administration and management in the United States, as the discussion there is specific to the complex situation in the country, resulting from historical developments, the dominant position of private health insurance, a debate about a single-payer system and the strongly divided political view about whether there should be at all any form of social health protection.

To the best of the authors’ knowledge no attempt has yet been made to classify administrative functions and provide systematic data on different schemes. A first discussion with colleagues from different SHI systems yielded the following preliminary data:

- *Germany* (126 SHI schemes, covering 70 million insured) has about 100,000 full-time equivalent (FTE) staff, with administrative cost ranging between 5 and 6% of revenues
- *The Netherlands* (covering 15.2 million) has about 7300 FTE with 4.4% admin cost.
- *Czechoslovakia* (8 insurers covering 10.6 million) has about 8500 FTE, administrative cost are between 2.9 and 3.5% of revenues.
- *The Philippines* (PhilHealth, covering about 70 million) has about 6000 FTE, and administrative cost are set at 8% of revenues.
- *Tanzania* (NHIF National Health Insurance Fund, covering about 2.5 million) has a total of 325 FTE.

These figures need to be read with extreme caution, but show a huge potential for asking questions about tasks, processes, efficiency and quality.

Key performance indicators for the implementation of social health insurance have been defined [4] and the need for professional health care management and administration has been recognized in developed countries [8]. A literature review identified seven major strategy areas potentially useful for improving performance among health care delivery organizations [3], but a detailed description of functional competencies and training needs for purchasing organizations has yet to be developed. In addition, service organization and governance are changing, leading to additional expectations that managers can (and will) accommodate to such changes and will become innovators themselves.

Moreover, indicators for administrative effectiveness, e.g. the number of staff per 1.000 insured, the time needed to get approval for services that require peer-approval (for example dental braces), the ease of process for a non-formally employed to enrol into the scheme, the time required to respond to a complaint, the effectiveness of a grievance processes, the availability of an ombudsman, amongst others need to be defined and the organization should be required to provide data to the public about such indicators, if only to show developments over time and possibly develop benchmarks and targets.

Anecdotal evidence suggests that some institutions have started internal re-organization and / or quality improvement initiatives, but usually with no formal methods to evaluate their impact or savings. Understanding the current costs and productivity of each administrative function relative to an organization’s peers (or the market) would help develop a baseline and influence informed decision making for planning, monitoring and evaluating investments in process efficiency and information technology (IT).

## Conclusion

Many countries (such as Ghana, Indonesia, Kazakhstan, Mongolia, Nepal to name but a few) transition from conceptual work and overall political decisions to the concrete set-up and development of a mandatory insurance / independent purchasing institution or work towards a more mature organization improving and streamlining structures and processes [13]. These countries would benefit from international benchmarks, an understanding of which qualifications are available on the labor market and which need to be specifically trained for and how much time and resources are required to setting up a functioning institution. A model scheme could be developed with indicative numbers of personnel in different departments, job descriptions and options for further development of such a scheme.

Such an institutional build-up could go hand-in-hand with strategies to increase efficiency, which are centred on simplifying and digitalizing procedures and optimising the size of administrative bodies to generate economies of scale [17]. Furthermore, regulatory changes might have a significant effect on administrative workload and costs of purchaser and providers.

We believe there is a strong case to be made for specific education and training in health management (for both purchasers and providers) in low- and middle-income countries. Health care management is a well-established discipline in developed countries with a wide range of training and research opportunities. Mature systems like for example in Germany, have over time developed their own apprenticeship structures to train specialized administrators in various social insurance schemes (social insurance clerk; in German *Sozialversicherungsfachangestellte/r*). More research and data, the development of model schemes and benchmarks, identifying good practices, and setting up international exchange and training opportunities would be valuable first steps. Such investments in building management and administration capabilities will be paid off as significant returns in accelerated effectiveness, efficiency and responsiveness of health care systems.

## Abbreviations

FTE: Full time equivalent
HRH_MA: Human resources for health in management and adminsitration
IT: Information technology
SHI: Social Health Insurance
UHC: Universal Health Care
WHO: World Health Organization
